# Self-construal priming modulates sonic seasoning

**DOI:** 10.3389/fpsyg.2023.1041202

**Published:** 2023-04-03

**Authors:** Jingxian Xu, Xiyu Guo, Mengying Liu, Hui Xu, Jianping Huang

**Affiliations:** ^1^Department of Psychology, Soochow University, Suzhou, China; ^2^School of Public Affairs, Zhejiang University, Hangzhou, China

**Keywords:** self-construal priming, emotional music, sensory marketing, sonic seasoning, tasting experience

## Abstract

**Introduction:**

“Sonic seasoning” is when music influences the real taste experiences of consumers. “Self-construal” is how individuals perceive, understand, and interpret themselves. Numerous studies have shown that independent and interdependent self-construal priming can affect a person's cognition and behavior; however, their moderating effect on the sonic seasoning effect remains unclear.

**Methods:**

This experiment was a 2 (self-construal priming: independent self-construal or interdependent self-construal) × 2 (chocolate: milk chocolate or dark chocolate) × 2 (emotional music: positive emotional music or negative emotional music) mixed design, and explored the moderating role of self-construal priming and the effect of emotional music on taste by comparing participants' evaluations of chocolates while listening to positive or negative music after different levels of self-construal priming.

**Results:**

After initiating independent self-construal, participants increased their ratings of milk chocolate sweetness when listening to music that elicited positive emotions, t(32) = 3.11, *p* = 0.004, Cohen's *d* = 0.54, 95% CI = [0.33, 1.61]. In contrast, interdependent self-construal priming led participants to perceive dark chocolate as sweeter when they heard positive music, t(29) = 3.63, *p* = 0.001, Cohen's *d* = 0.66, 95%CI = [0.44, 1.56].

**Discussion:**

This study provides evidence for improving people's individual eating experience and enjoyment of food.

## Introduction

Researchers have been increasingly interested in sonic seasoning in recent decades (Knöferle and Spence, 2012; Spence, 2017; Spence et al., 2019; Spence and Di Stefano, 2022). This term refers to the deliberate matching of music with flavor to enhance the multisensory tasting experience (Sedacca, 2016). For instance, music has been composed for specific flavors such as “sweet,” “bitter,” or “salty” based on the flavors of the food (Crisinel and Spence, 2010). Wang et al. (2017) indicated that spiciness was associated with auditory attributes of high pitch, fast tempo, and high levels of distortion. Wang et al. (2021) further explored the acoustical/musical attributes that best match saltiness and found that auditory attributes based on emotional associations (negative valence, minor mode, and high arousal), long decay durations, regular rhythm, and a high degree of auditory roughness were associated most strongly with saltiness. These findings on sonic seasoning have been used in marketing-led activations (Spence et al., 2021) and further understanding of how music influences consumers' perceptions of food. Crisinel et al. (2012) demonstrated for the first time that individuals evaluate food differently based on the music they hear while tasting it. Participants were asked to rate the taste of several bittersweet toffees while listening to sweet or bitter music. It was found that when they listened to the sweet soundtrack, the sweetness of the toffee was evaluated higher than when listening to the bitter soundtrack. Wang et al. (2020) added that chocolate ratings could be affected only when participants listened to sweet or bitter music before or during a chocolate tasting.

Other previous literature also affirms that when music is playing in the background while people eat, the emotions that individuals experience from or associated with the music are transferred to the tasting experience itself, where these emotions are considered to act as an important mediator during sonic seasoning demonstrations (Kantono et al., 2016, 2019; Reinoso-Carvalho et al., 2019, 2020). For instance, North (2012) showed that emotional connotations associated with background music influenced consumers' taste perception of wine. In addition, Kantono et al. (2016) showed that music evoked positive emotions (satisfaction, happiness, and amusement) that influenced the perceived sweetness of gelati, while music evoked negative emotions (contempt, disappointment, and disgust) that influenced the perceived bitterness of gelati. Reinoso-Carvalho et al. (2019) discovered that compared to music associated with positive emotion when participants listened to music associated with negative emotion, they rated the same beer as more bitter, with higher alcohol content, and were willing to pay more for the beer. The music that evokes positive emotions is often performed with high intensity, a brighter timbre, staccato articulation, and a rapid tempo. Conversely, music that evokes negative emotions is often performed with low intensity, a duller timbre, legato articulation, and a slow tempo (Quinto and Thompson, 2013).

Research showed that auditory information like music could impact the subjective evaluation of the taste of food (Spence and Di Stefano, 2022). For example, Reinoso-Carvalho et al. (2020) discovered that emotional music (i.e., music that evokes positive or negative emotions), as opposed to crossmodal music (i.e., soft or hard music; the former means imaging the music is consistent with the smooth food texture, the latter refers to imaging the music is consistent with the rough food texture), had a more prominent effect on food flavor and purchase intention. Specifically, participants rated the chocolate as sweeter, and the purchase intention was higher when music judged to elicit positive valence was played compared to music that was judged to elicit negative valence. However, the same chocolate was judged to be more bitter when exposed to music that conveyed negative rather than positive emotions. The researchers proposed that the sweeter (bitter) taste of chocolate induced by positive (negative) music could be explained by a theory similar to the “attention-shifting or redirection effect” (Johnson and Proctor, 2004). That is music that transmits positive or negative emotions can allow participants to transfer their feelings to the flavor perception of chocolate, which in turn affects their purchase intention. An analogous conclusion was proposed in the study by Ziv (2018) that participants generally considered cookies to taste better when listening to pleasant music.

As described above, people's perception of food is affected by contextual factors. Moreover, a large number of studies have suggested that individuals from different cultural backgrounds are influenced by context to different degrees (Wan et al., 2014; Jeong and Lee, 2021), which may be attributed to differences in cognitive style (Henrich, 2014). As one of the important cultural characteristics, self-construal is often used to explain cultural differences in human behavior, cognition, and emotion. It is primarily about how individuals perceive, understand, and interpret themselves. Markus and Kitayama (1991) suggested that independent self-construal is more predominant in Western culture, which conceptualizes the self as an autonomous and bonded entity, emphasizing self-independence and uniqueness. By contrast, interdependent self-construal is more predominant in East Asian culture, which conceptualizes the self as interconnecting and overlapping with others, emphasizing the importance of living in harmony with other groups and individuals. In general, interdependent self-construal facilitates the spontaneous association of a focal object with context compared to independent self-construal (Goh et al., 2007). Moreover, interdependent self-construal consumers believe that a higher price means a higher quality than independent self-construal consumers because the former are more susceptible to perceiving the association between product elements (Lalwani and Shavitt, 2013).

While self-construal can be judged by scales (Singelis, 1994) or inferred based on an individual's ethnicity (Van Baaren et al., 2003), researchers prefer to manipulate self-construal through priming methods when studying it in a laboratory (Kühnen and Oyserman, 2002; Reinoso-Carvalho et al., 2020). In particular, participants were asked to search first-person pronouns such as “I” or “mine” in a story to represent independent self-construal, while “we” or “our” imply interdependent self-construal. Considering the container that holds the food as a background, Huang et al. (2021) suggested that self-construal priming could modulate the influence of receptacles on food perception. Compared to independent self-construal priming, interdependent self-construal priming elicited a greater influence of the size of the plate on participants' willingness to pay (WTP) for noodles and the pleasantness ratings of the noodles served on the red plate. Therefore, considering emotional music as a contextual factor, it is reasonable to expect that self-construal priming could moderate the effect of emotional music on food perception.

This study investigates whether self-construal can modulate the effects of emotional music on food perception. Owing to how interdependent self-construal can improve the connection between the target and the contextual background (Masuda and Nisbett, 2001; Goh et al., 2007) and influence object processing that is dependent on context (Kühnen and Oyserman, 2002), we hypothesized that the participants' perception of food flavor would not be influenced by the background music under independent self-construal priming. Conversely, different emotional music would affect participants' evaluation of food after initiating the interdependent self-construal.

## Methods

### Participants

To estimate the sample size, we used the Easypower package (McGarvey, 2015) in R.4.2.0 (R Core Team, 2022) to conduct an *a priori* power analysis. According to the mixed design of 2 (self-construal priming: independent self-construal or interdependent self-construal) × 2 (chocolate: milk chocolate or dark chocolate) × 2 (emotional music: positive emotional music or negative emotional music). The sample size should not be < 48 with an effect size of the quadruple interaction of 0.25, a statistical power of 0.95, and an alpha of 0.05. Considering the balance between different experimental conditions, a total of 141 college students from Soochow University took part in the experiment. However, we had to exclude the data of 13 participants from analyses because their answers to all the questions were the same. Therefore, 128 valid data were received (M_age_ = 20.12 years, SD_age_ = 0.16 years, ranging from 18 to 26 years; 35 males). All participants were right-handed and had normal or corrected eyesight, without color blindness or color weakness. All participants were paid 10 Chinese yuan after completing the experiment.

### Apparatus and materials

The experiment used a 27-inch monitor with a resolution of 2,560 × 1,440 pixels and a refresh rate of 60 Hz. The online questionnaire was based on the Qualtrics platform (https://www.qualtrics.com), including basic information (e.g., sex, age, and degree of hunger) and the priming of self-construal, which refers to the study by Sui and Han (2007). We measured the degree of hunger based on previous literature that focused on food and/or beverages (Biswas et al., 2021; Moss and McSweeney, 2021) because hunger could potentially influence taste perceptions (Hanci and Altun, 2016). Participants were asked to carefully read a travel story that was presented randomly and then click on all the personal pronouns in the story (Grossmann and Jowhari, 2018).

In line with Reinoso-Carvalho et al. (2020), our study used milk chocolate (Callebaut N. 823, containing milk and at least 33.6% cocoa solids) and dark chocolate (Callebaut N. 811, containing no milk and at least 54.5% cocoa solids), as well as positive and negative emotional music from https://tinyurl.com/music-emotions-xcultural. Specifically, the negative emotional music sample could evoke more negative valence, and the positive emotional music sample could evoke more positive valence. The chocolate rating task consisted of the following four questions (7-point Likert scale): “What do you think of the sweetness of the chocolate?” “What do you think of the bitterness of the chocolate?” “How much do you like the chocolate in the experiment?” and “How likely are you to purchase the chocolate?” In addition, they were required to answer how much they would be willing to pay for a bar of chocolate in RMB (Renminbi, Chinese Yuan). The Chinese revision of the Positive and Negative Affect Scale (PANAS; Watson et al., 1988) developed by Qiu et al. (2008) was applied to determine participants' emotions. Specifically, participants had to rate on a 5-point scale positive (e.g., gratitude, energetic, active, cheerful, excited, enthusiastic, proud, happy, and joyful) or negative emotional words (e.g., scared, afraid, guilty, ashamed, nervous, irritable, angry, jittery, and sad) that appeared randomly. Scores from 1 to 5 represent very slightly or not at all, a little, moderately, quite a bit, and extremely, respectively.

An online pretest was conducted to ensure that the emotional music samples were effective (the negative emotional music sample evoked more negative valence, and the positive emotional music sample evoked more positive valence). A total of 34 participants (eight men and 26 women) between the ages of 19 and 25 years (M = 21.26 years, SD = 1.58) were recruited. Participants were required to listen to the emotional music sample and complete the Chinese PANAS mentioned earlier. The number of participants who listened to the positive music emotional sample is equal to the number of participants who listened to the negative emotional music sample. The results showed that participants rated significantly higher on the Positive (M = 2.30, SD = 0.87) than Negative Affect Scale (M = 1.29, SD = 0.35) after listening to the positive emotional music sample, *t*_(16)_ = 4.34, *p* = 0.001, Cohen's *d* = 1.05, 95% CI = [0.51, 1.50]. As expected, participants rated significantly higher on the Negative (M = 2.66, SD = 0.47) than Positive Affect Scale (M = 1.63, SD = 0.47) after listening to the negative emotional music sample, *t*_(16)_ = 6.52, *p* < 0.001, Cohen's *d* = 1.58, 95% CI = [0.69, 1.36]. These results indicated that the positive emotional music sample we used in this experiment could evoke more positive valence while the negative emotional music sample could evoke significantly more negative valence.

### Design

This experiment was a 2 (self-construal priming: independent self-construal or interdependent self-construal) × 2 (chocolate: milk chocolate or dark chocolate) × 2 (emotional music: positive emotional music or negative emotional music) mixed design. Self-construal priming and chocolate were between-subject variables, and emotional music was a within-subject variable. The type of chocolate was randomly matched with the two pieces of music. The dependent variables were the rating of the sweetness, bitterness, liking, WTP, and purchase intention of the chocolate that they ate. Each participant ate no more than two pieces of chocolate twice.

### Procedure

Participants were first asked to fill in personal information and complete the self-construal priming task. Then they had to complete the PANAS as baseline emotion. After that, participants rinsed their mouths with water and put on headphones. It should be noted that although the duration of positive and negative music was inconsistent (69 vs. 60 s), the volume of the two kinds of music was controlled at 70 ± 6 dB. The experiment requested participants to taste a bar of chocolate on a plate we had prepared while listening to a random type of music played through headphones. Meanwhile, they were allowed to continue savoring a second of the same chocolate if they had finished tasting the first piece before the end of the music. Next, participants were required to evaluate the chocolate they ate in the five dimensions mentioned above and accomplish the PANAS again. After rinsing their mouths again with water, participants completed a similar task of matching another type of music with another type of chocolate. All the analysis data are publicly available from the Open Science Framework repository (OSF) at https://osf.io/qhdgz/.

## Results

Before formal analysis, we first conducted a manipulation check on the emotional scores of the participants after listening to positive and negative emotional music. As expected, music that conveyed positive emotions induced significantly higher positive (M = 2.25, SD = 0.83) than negative feelings (M = 1.15, SD = 0.33), *t*_(127)_ = 14.46, *p* < 0.001, Cohen's *d* = 1.28, 95% CI = [0.95, 1.25]. Surprisingly, the participants also scored significantly higher on the Positive (M = 1.86, SD = 0.77) than Negative Affect Scale (M = 1.44, SD = 0.51) after listening to negative emotional music, *t*_(127)_ = 4.74, *p* < 0.001, Cohen's *d* = 0.42, 95% CI = [0.24, 0.59]. We further analyzed the emotional scores of the participants after listening to positive and negative emotional music. The results showed that positive emotional music (M = 2.25, SD = 0.83) evoked significantly more positive valence than negative emotional music (M = 1.86, SD = 0.77), *t*_(127)_ = 6.02, *p* < 0.001, Cohen's *d* = 0.53, 95% CI = [0.27, 0.53], while negative emotional music (M = 1.44, SD = 0.51) scored significantly higher negative valence than positive emotional music (M = 1.15, SD = 0.33), *t*_(127)_ = 6.50, *p* < 0.001, Cohen's *d* = 0.57, 95% CI = [0.20, 0.38].

**Figure 1:**
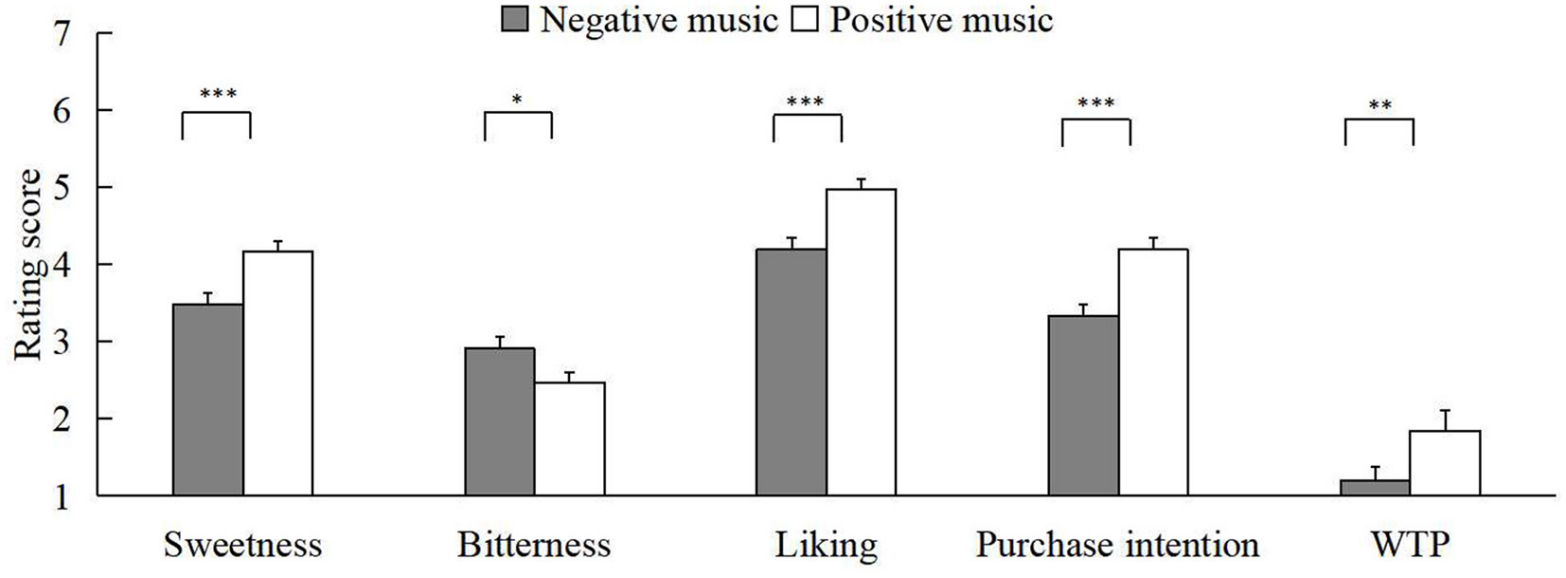
The main effect of emotional music on the five dimensions. Note that error bars show the standard errors of the means, *p < 0.05, **p < 0.01. and ***p < 0.001.

**Figure 2:**
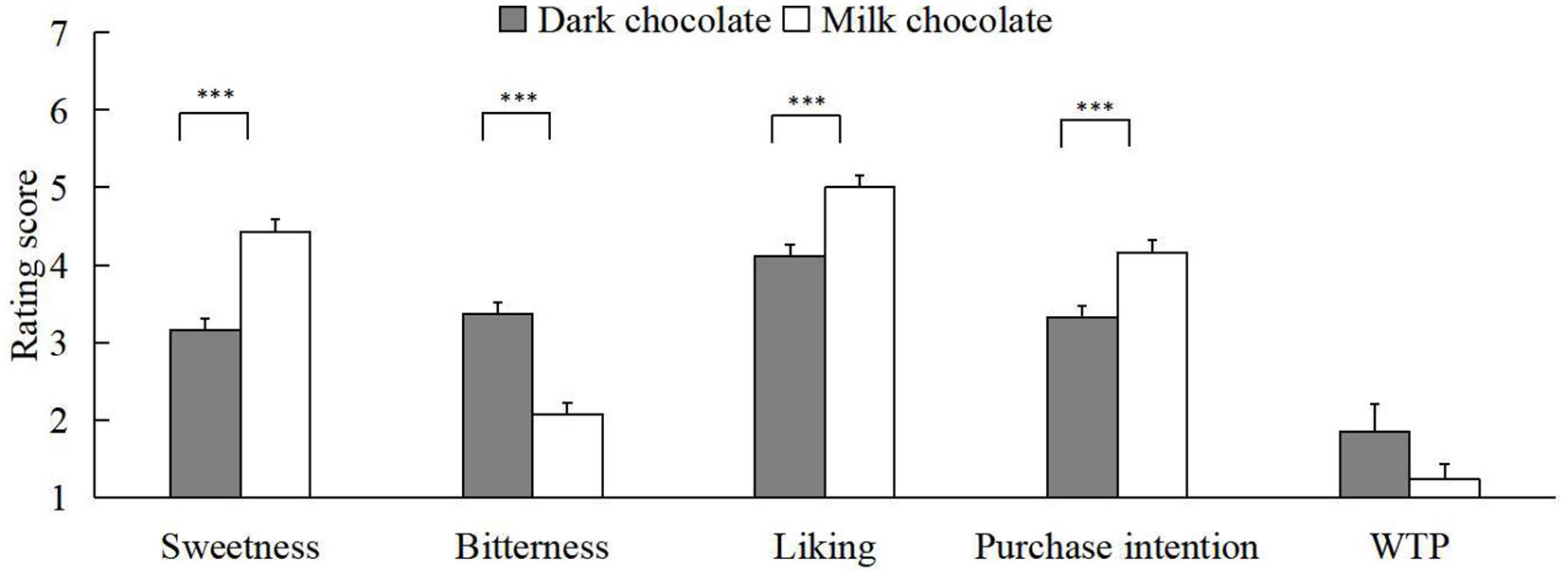
The main effect of chocolate is on the five dimensions. Note that error bars show the standard errors of the means, ***p < 0.001.

We performed a 2 (self-construal priming: independent self-construal or interdependent self-construal) × 2 (chocolate: milk chocolate or dark chocolate) × 2 (emotional music: positive emotional music or negative emotional music) repeated-measures ANOVA on valid data. The results found that the main effect of emotional music was significant in all five aspects of chocolate evaluation [*F*_sweetness(1, 124)_ = 21.92, *p* < 0.001, $\eta_{p}^{2}$ = 0.15; *F*_bitterness(1, 124)_ = 6.91, *p* = 0.010, $\eta_{p}^{2}$ = 0.05; *F*_liking(1, 124)_ = 21.62, *p* < 0.001, $\eta_{p}^{2}$ = 0.15; *F*_purchaseintentions(1, 124)_ = 24.97, *p* < 0.001, $\eta_{p}^{2}$ = 0.17; *F*_WTP(1, 124)_ = 10.43, *p* = 0.002, $\eta_{p}^{2}$ = 0.08]. As shown in Figure 1, compared to negative music, the participants rated the chocolate as having higher sweetness (M_1_ = 3.48, SD_1_ = 1.68; M_2_ = 4.16, SD_2_ = 1.54), liking (M_1_ = 4.19, SD_1_ = 1.72; M_2_ = 4.97, SD_2_ = 1.43), and lower bitterness (M_1_ = 2.91, SD_1_ = 1.80; M_2_ = 2.47, SD_2_ = 1.56) when the emotional music was positive. In addition, the purchase intentions (M_1_ = 4.19, SD_1_ = 1.67; M_2_ = 3.34, SD_2_ = 1.63) and WTP (M_1_ = 1.84, SD_1_ = 2.97; M_2_ = 1.20, SD_2_ = 1.93) for chocolate were further intensive when participants were exposed to positive rather than negative music. The experimental results also revealed that the type of chocolate had a significant main effect on the sweetness [*F*_(1, 124)_ = 33.18, *p* < 0.001, $\eta_{p}^{2}$ = 0.21], bitterness [*F*_(1, 124)_ = 36.46, *p* < 0.001, $\eta_{p}^{2}$ = 0.23], liking [*F*_(1, 124)_ = 17.45, *p* < 0.001, $\eta_{p}^{2}$ = 0.12], and purchase intention [*F*_(1, 124)_ = 13.16, *p* < 0.001, $\eta_{p}^{2}$ = 0.10] of chocolate (as shown in Figure 2). Specifically, participants considered milk chocolate to be sweeter (M_dark_ = 3.16, SD_dark_ = 1.13; M_milk_ = 4.43, SD_milk_ = 1.32) and less bitter (M_dark_ = 3.37, SD_dark_ = 1.19; M_milk_ = 2.07, SD_milk_ = 1.24) than dark chocolate. Then, they preferred milk chocolate (M_dark_ = 4.11, SD_dark_ = 1.09; M_milk_ = 5.00, SD_milk_ = 1.27) and more purchase intentions for it (M_dark_ = 3.33, SD_dark_ = 1.15; M_milk_ = 4.16, SD_milk_ = 1.39) compared to dark chocolate.

**Figure 3:**
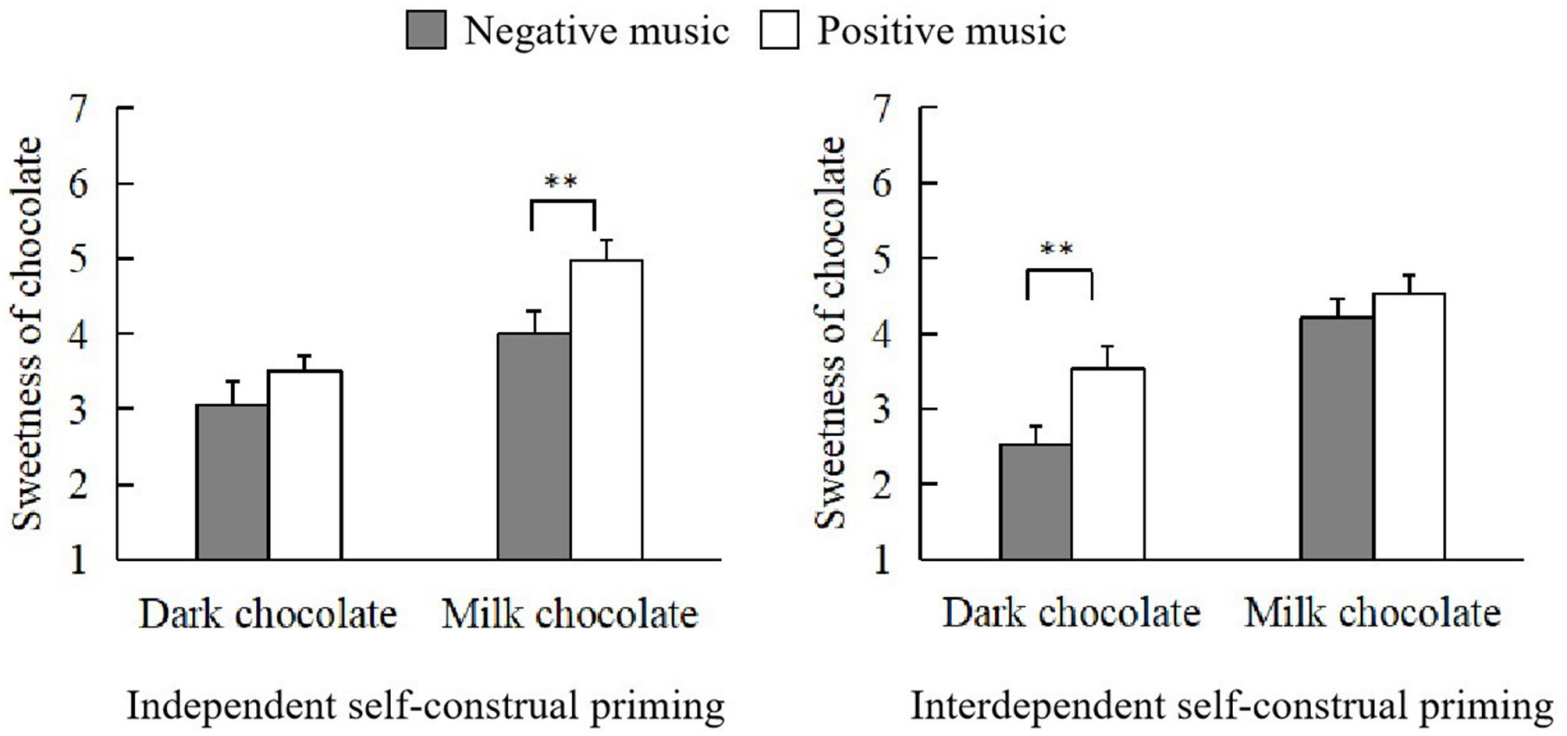
Triple interaction effect among emotional music, self-construal priming, and chocolate on the sweetness of chocolate. Note that error bars show the standard errors of the means, **p < 0.01.

The results demonstrated that there was a significant triple interaction among emotional music, self-construal priming, and chocolate on the sweetness of chocolate, *F*_(1, 124)_ = 4.15, *p* = 0.04, $\eta_{p}^{2}$ = 0.03. Based on this, we further analyzed the effects of self-construal priming and chocolate type on the evaluation of chocolate sweetness under two music conditions. As shown in Figure 3, positive music improved the sweetness of milk chocolate (M = 4.97, SD = 1.53) more than negative music (M = 4.00, SD = 1.75) when independent self-construal was primed, *t*_(32)_ = 3.11, *p* = 0.004, Cohen's *d* = 0.54, 95% CI = [0.33, 1.61]. However, music with positive emotions increased the sweetness of dark chocolate (M = 3.53, SD = 1.57) more than music with negative emotions (M = 2.53, SD = 1.25) under the priming of interdependent self-construal, *t*_(29)_ = 3.63, *p* = 0.001, Cohen's *d* = 0.66, 95% CI = [0.44, 1.56].

## Discussion

This study aimed to explore the moderating role of different types of self-construal in the process of emotional music affecting food evaluation. The experimental results mainly suggested the following three points. First, positive music can improve participants' ratings of chocolate and purchase intention. Consistent with previous studies (Ziv, 2018; Kantono et al., 2019; Reinoso-Carvalho et al., 2019, 2020), our study elucidated that participants find chocolate to be sweeter after listening to positive music, which in turn increases their liking and purchase intention for it. Skaczkowski et al. (2016) argued that positive (negative) emotions evoked by positive (negative) music could be transferred to individuals' authentic tasting experience based on sensation transference effects.

Second, our results demonstrated that self-construal priming could modulate sonic seasoning. In particular, music with a positive emotion was more likely to enhance the participants' evaluation of the sweetness of milk chocolate under the priming of independent self-construal and dark chocolate under the priming of interdependent self-construal. However, independent self-construal priming did not influence participants' evaluation of dark chocolate sweetness. As researchers have suggested before, individuals with independent self-construal tend to adopt an analytic thinking style that emphasizes the independence of individual objects, whereas individuals with interdependent self-construal tend to adopt a holistic style of thinking emphasizing that the world is composed of interrelated elements (Nisbett et al., 2001; Monga and John, 2007, 2008). This may explain why different self-construal priming types have different influences on sonic seasoning. The self-construal of interdependence can guide the participants to adopt a holistic way of thinking to perceive the relationship between the background music and dark chocolate, which leads to the influence of positive music on the evaluation of dark chocolate's sweetness. However, an analysis of milk chocolate showed that the evaluation of the sweetness of milk chocolate was not affected by emotional music under the initiation of the self-construal of mutual dependence. The possible reason is that milk chocolate itself has been perceived as sweet, so there is no significant difference in the evaluation of sweetness between positive and negative music, even though the participants were under the priming of interdependent self-construal.

Previous studies have focused on the sonic seasoning of food by individuals from different countries (Reinoso-Carvalho et al., 2019, 2020) or on explaining the influence of self-construal priming on food perception (Hansen, 2019; Huang et al., 2021). The innovation of this study is to make a thorough inquiry into how self-construal priming impacts the relationship between music-evoked emotion and the perception of taste. Theoretically, by comparing independent and interdependent self-construal priming on food evaluations, the present study reveals the moderating role of self-construal priming types on the effect of emotional music on real food tasting, which could enrich the findings of sonic seasoning. For the marketing field, our study provides a certain theoretical reference for improving individual diet experience and boosting happiness in eating. In particular, marketers can control consumers' perception of flavors in food or beverage products by playing different styles of music according to their cultural background. In addition, it is a bright prospect for marketers to adopt words that represent different self-construals on the packaging of food to change consumers' cognition.

Like other studies, this study has unavoidable limitations. First, after listening to the negative music, participants did not report a higher negative valence than positive valence. There were several possible explanations. First, a previous study found that compared to an audio-only presentation, an audio-visual congruent presentation, which represented congruent audio and visual emotions (e.g., happy face and happy music), could lead to a more intense emotional response. This effect occurred in both positive and negative music, and the effect was larger for positive music (Pan et al., 2019). The baseline emotional score in our study showed that participants were significantly more positive before the experiment. Thus, it was possible that the negative emotional music was not capable of evoking a strong negative emotional response to make negative emotion dominant for the participants. Moreover, people listened to music while eating chocolate and then completed the PANAS, which might result in emotional scores that not only represent the valence evoked by the music but also the taste of chocolate. As a result, future experiments should measure participants' positive or negative valence to the music itself using the GEMS (Zentner et al., 2008) or GEMIAC questionnaire (Coutinho and Scherer, 2017) and evaluate the music with respect to different taste categories. In the current study, we focused on the effects of music that can induce positive or negative emotions on food perception. Although we can see the effects of the music on the five dimensions and between independent and interdependent self-construal priming we cannot gauge the extent to which these effects are significant because there was no control (i.e., a non-music condition) for comparison. As a result, future experiments could add a non-music condition.

Second, the participants in this study were university students aged ~20 and were Chinese. We are not sure whether the findings of the study apply to people of other ages and cultures. Reinoso-Carvalho et al. (2020) found that the sonic seasoning effects were different on people from LATAM and Asia. Moreover, previous studies have found that sex differences in self-construal vary across different nations (Costa et al., 2001; Schmitt et al., 2008). Future experiments could expand the sample to different cultures and ages.

Third, this study showed that self-construal could modulate the effects of emotional music on food perception. Previous research discovered that crossmodal congruency between music and food/beverage had effects on the tasting experience (Wang and Spence, 2015; Reinoso-Carvalho et al., 2020; Motoki et al., 2022). For example, Wang and Spence (2015) demonstrated that the music chosen to be congruent with each wine was indeed rated a better match than other pieces of music, and the music significantly influenced the perceived acidity and fruitiness of the wine. Future experiments could investigate whether self-construal could modulate the effects of crossmodally congruent music on food perception.

Finally, Mawad et al. (2015) found that field-independent and field-dependent tendencies affected the visual attention processing of yogurt label information. Individuals with an independent tendency would pay more attention to the health label information of yogurt. It is well-known that individuals with field-independent tendencies are more detached from their surroundings and less susceptible to external cues than field-dependent individuals. Likewise, individuals who have experienced independent self-construal were also more focused on themselves. Therefore, we can further explore the influence of music on the process of an individual's attention to food and whether self-construal still modulates this process in future studies. Moreover, Peng-Li et al. (2020) discovered through eye tracking that sweet music improved gaze time to sweet food, while salty music increased their fixation time on salty food. Notably, Peng-Li et al. (2020) demonstrated the influence of different music on food with different flavors based on the cross-modality association of music and flavor. In light of this, follow-up studies should further examine the moderating role of self-construal in the effect of emotional music on an individual's attention to sweet or bitter foods.

## Conclusion

The results of this study suggest that self-construal priming has a moderating role in how emotional music affects the authentic flavor experience. For example, under independent self-construal priming, there was no difference in the evaluation of the sweetness of dark chocolate between positive and negative music. However, positive music significantly improved participants' evaluation of the sweetness of milk chocolate. In contrast, interdependent self-construal priming obtained the converse result. Compared with negative music, positive music can enhance the sweetness of dark chocolate, but neither kind of music influences the sweetness of milk chocolate. This study provides evidence that could improve an individual's eating experience by promoting happiness when eating.

## Data availability statement

The original contributions presented in the study are included in the article/Supplementary material, further inquiries can be directed to the corresponding authors.

## Ethics statement

The studies involving human participants were reviewed and approved by Human Research Ethics Committee of the Department of Psychology of Soochow University. The patients/participants provided their written informed consent to participate in this study. Written informed consent was obtained from the individual(s) for the publication of any potentially identifiable images or data included in this article.

## Author contributions

JX: methodology, investigation, data curation, and formal analysis. XG: formal analysis, methodology, writing, and original draft preparation. ML: writing, review, and editing. HX: supervision, writing, review, and editing. JH: conceptualization, supervision, writing, review, and editing. All authors contributed to the article and approved the submitted version.
